# Novel *in situ* visualisation of rat intestinal absorption of polyphenols via matrix-assisted laser desorption/ionisation mass spectrometry imaging

**DOI:** 10.1038/s41598-019-39405-w

**Published:** 2019-02-28

**Authors:** Huu-Nghi Nguyen, Mitsuru Tanaka, Baorui Li, Tomoya Ueno, Hideki Matsuda, Toshiro Matsui

**Affiliations:** 1 Division of Bioscience and Bioenvironmental Sciences, Faculty of Agriculture, Graduate School of Kyushu University, 744 Motooka, Nishi-ku, 819-0395 Fukuoka, Japan; 2 Division of Research and Development, Yaizu Suisankagaku Ind. Co. Ltd., 5-8-13 Kogawashinmachi, Yaizu, Shizuoka 425-8570 Japan

## Abstract

Matrix-assisted laser desorption/ionisation mass spectrometry imaging (MALDI-MSI) is presently used in physiological evaluations for visualisation of targets in organs. In the present study, MALDI-MSI was used as a visualisation technique to investigate the intestinal absorption of polyphenols. Nifedipine/phytic acid-aided MALDI-MSI was performed to visualise theaflavin-3′-*O*-gallate (TF3′G) and epicatechin-3-*O*-gallate (ECG) in the rat jejunum for 50-µM, 60-min transport experiments. Non-absorbable TF3′G was successfully visualised at the apical region, whereas absorbable ECG was detected throughout the rat jejunum. MALDI-MSI was also performed to determine the transport routes of the target metabolites. Signals corresponding to TF3′G and ECG in the membranes were diminished following treatment with inhibitors targeting the monocarboxylic acid transporter and organic anion transporting polypeptides. Enhanced visualisation of TF3′G was achieved by inhibiting efflux routes. Our findings demonstrated that the present MALDI-MSI can provide critical spatial informations on intestinal absorption of targets, by which TF3′G and ECG were incorporated into intestinal tissues, followed by efflux back to the apical compartment. In addition, MALDI-MSI analyses suggested that TF3′G was resistant to phase II metabolism during the influx/efflux processes, whereas ECG was susceptible to methylation and sulphation reactions. In conclusion, inhibitor-aided MALDI-MSI could serve as a powerful *in situ* visualisation technique for verifying intestinal transport routes and investigating the metabolism of penetrants.

## Introduction

Matrix-assisted laser desorption/ionsation mass spectrometry imaging (MALDI-MSI) is a promising *in situ* analytical tool that can be used in pharmacological studies. MALDI-MSI allows simultaneous visualisation of drugs or food compounds, such as polyphenols, peptides, proteins, lipids, and their metabolites, in the organs^1–6^. A major advantage of MALDI-MSI is that it allows direct visualisation of the spatial localisation of analytes in the target tissues. Furthermore, MSI can be used to visualise not only analytes, but also their metabolites, in a single assay without the need for antibodies, which are required for immune staining visualisation^2,5,6^. Therefore, the currently proposed MSI technique offers a wide range of analytical applications for studying the absorption of food compounds, since some compounds may undergo degradation and/or become subjected to phase II metabolism during the intestinal absorption process^2,7,8^.

Polyphenols (e.g., hesperidin) have been reported to show various pharmacological effects such as: anti-hypertensive^9^, anti-diabetic^10^ and anti-inflammatory effects^11^, while hesperidin was metabolised to form its aglycon, hesperetin, or in most cases hesperetin conjugates during intestinal absorption process^12^. However, crucial absorption/metabolic processes including transport routes of polyphenols in the intestine have not been fully clarified yet. In the present study, tea polyphenols, epicatechin-3-*O*-gallate (ECG) and theaflavin-3′-*O*-gallate (TF3′G), were selected as the targets to demonstrate the advantages of MALDI-MSI for intestinal absorption studies. ECG is a typical catechin that has been reported to be absorbed across the intestinal membrane^13^. TF3′G is a condensed catechin that is formed with ECG and epicatechin and cannot be absorbed across the intestinal membrane^14^. In a previous study using Sprague-Dawley (SD) rats, ECG was shown to be absorbed in the blood after a single oral administration. ECG remained intact, and sulphated ECG forms were detected^15,16^. In addition, experiments using Caco-2 cells showed that ECG was transported via the monocarboxylic acid transporter (MCT) route^17^. In our previous absorption study on theaflavins (TFs) in 60-min-Caco-2 cell transport, TFs were not detected on the basolateral side by liquid chromatography-time-of-flight-mass spectrometry (LC-TOF-MS)^18^. Regardless of their non-absorption properties, TFs were found to play physiological roles in the regulation of intestinal transport routes. In particular, TF3′G was suggested to suppress the expression of intestinal peptide transporter 1 (PepT1) and upregulate the expression of the tight junction (TJ) proteins in Caco-2 cells via the activation of AMP-activated protein kinase (AMPK)^18,19^. Although these findings led to a speculation that TFs must exert physiological function in the intestinal tissue, it remains unclear whether TFs could enter the intracellular side of the intestinal membrane. Considering the aforementioned advantages of MALDI-MSI, we attempted to apply the MSI-guided visualisation technique to obtain direct evidence for the location, absorption route(s), and metabolism of penetrants during absorption. To visualise the *in situ* absorption behaviour of polyphenols using MALDI-MSI, nifedipine was used as the preferred matrix reagent, together with other common matrix reagents. Our previous studies showed that nifedipine enhanced the ionisation of less-ionisable polyphenols (e.g., flavonols, flavones, flavanones, flavonones, chalcones, stilbenoids, and phenolic acids), by removal of a proton from the polyphenol skeleton due to its photobase properties^20^.

## Results

### Detection of TF3′G and ECG in the rat jejunum membrane using MALDI-MSI

**Figure 1:**
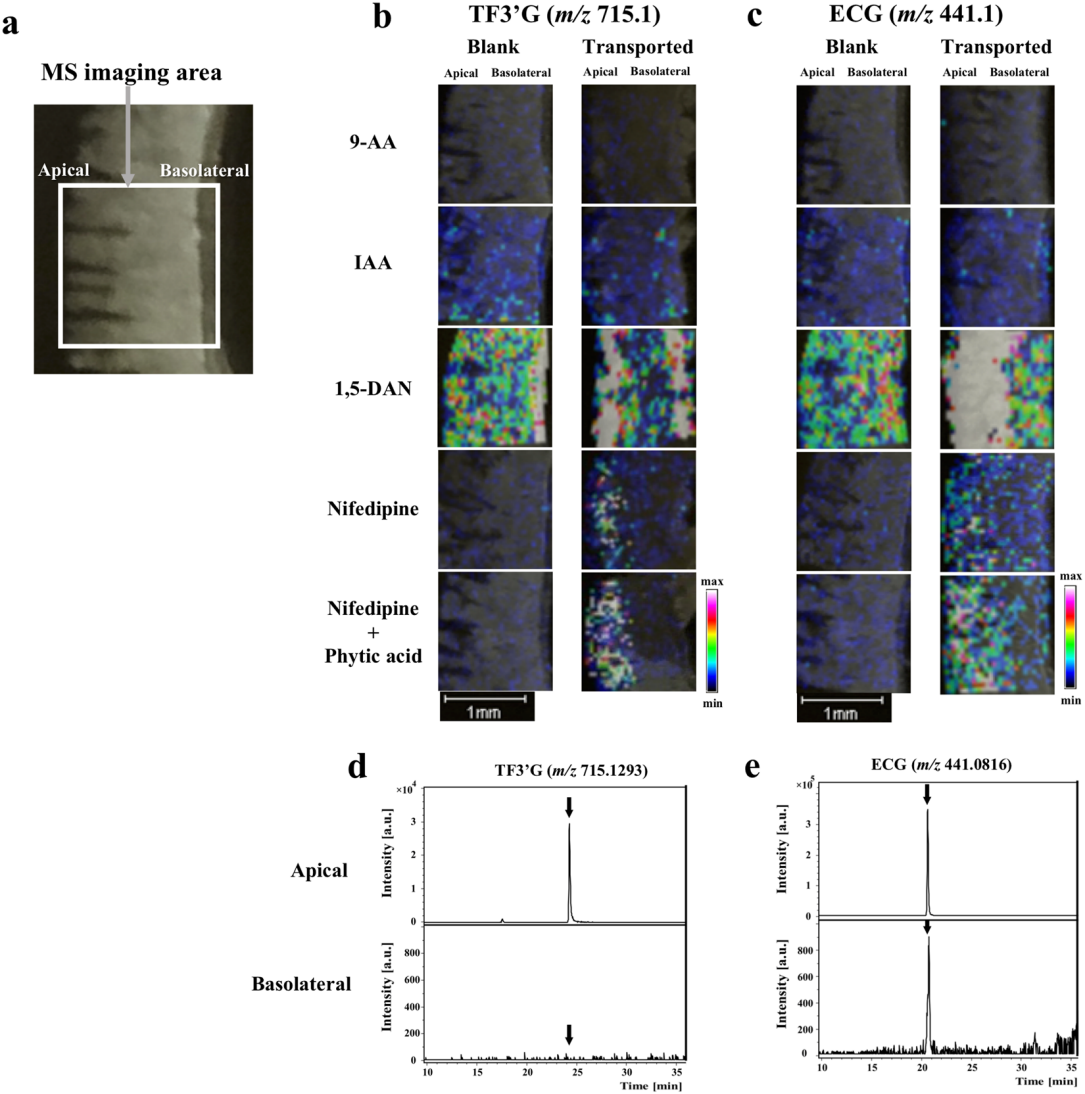
MALDI-MSI-based detection of TF3′G and ECG in the SD rat jejunum membranes. Representative optical image of the rat jejunum membrane (**a**), distribution of TF3′G (**b**) and ECG (**c**) in the membranes and LC-TOF-MS chromatograms of TF3′G (**d**) and ECG (**e**) in the apical and basolateral solutions after 50 µM, 60-min transport experiments in the Ussing Chamber system. Matrix reagents, including 9-AA (10 mg/mL), IAA (20 mg/mL), 1,5-DAN (20 mg/mL), nifedipine (20 mg/mL), and nifedipine (20 mg/mL) containing 5 mM phytic acid in acetonitrile/water (3:1, v/v), were sprayed individually onto the sections mounted onto ITO glass slides. TF3′G (*m/z* 715.1) and ECG (*m/z* 441.1) were visualised via MALDI-MSI in the negative ion-linear mode at the spatial resolution of 50 µm. Intensity signals corresponding to TF3′G and ECG are shown as fixed pseudocolour scales. LC separations were performed on a Cosmosil 5C_18_-MS-II column (2.0 mm × 150 mm) and eluted for 30 min with 0% to 100% MeOH/FA (100/0.1, v/v). MS conditions are described in the Methods section.

To obtain high-intensity MALDI-MS signals from the TF3′G and ECG targets in their transported intestinal membranes, matrix reagents that were reported to be suitable for polyphenols^2,20–22^ were selected for the present MALDI-MSI experiments. SD rat jejunum membranes subjected to 60-min transport experiments for both polyphenols (50 µM) were used for this experiment. Figure 1b,c show that both targets were successfully detected and visualised in the negative ion mode ([M-H]^−^: ECG, *m/z* 441.1; TF3′G, *m/z* 715.1) using 1,5-diaminonaphthalene^2^ (1,5-DAN, 20 mg/mL) and nifedipine^20^ (20 mg/mL). In contrast, TF3′G and ECG were not detected using 9-aminoacridine^21^ (9-AA, 10 mg/mL) and *trans*-3-indoleacrylic acid^22^ (IAA, 20 mg/mL). Although 1,5-DAN showed strong visualisation intensities for both polyphenols, unexpected high background detection in the blank section of TF3′G (Fig. 1b) impaired the highly selective visualisation of the analytes in the intestinal membrane sections. Thus, additional MALDI-MSI experiments were performed using nifedipine as the matrix reagent; nifedipine was found to be suitable for TF3′G and ECG visualisation. Considering the effect of contaminating minerals present in the tissues on MALDI-ionisation of targets, 5 mM phytic acid, which can enhance the MALDI-ionisation by chelating minerals such as Na^+^ and K^+^,^4^ was added to the nifedipine matrix solution to enhance the visualisation of the spatial distribution of both analytes (Fig. 1b,c).

The optimised nifedipine/phytic acid-aided MALDI-MSI method (Fig. 1b,c) also showed that TF3′G was located at the apical region in 60-min transported rat jejunum membranes, whereas ECG was detected throughout the membrane. LC-TOF-MS did not detect TF3′G (Fig. 1d) but detected ECG (Fig. 1e) in the basolateral solution after 60-min transport experiments. Thus, the established MSI method could serve as a powerful *in situ* tool to validate the absorbability of target compounds across the intestinal membrane.

### Determination of the absorption routes of TF3′G and ECG in the rat jejunum membrane using MALDI-MSI

**Figure 2:**
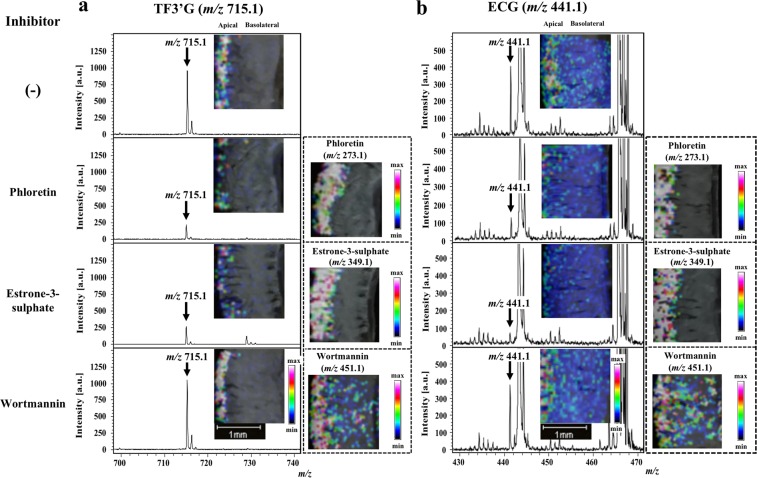
MALDI-MSI visualisation of TF3′G and ECG in jejunum membranes subjected to 60-min transport experiments in the absence or presence of influx transport inhibitors. Phloretin (200 µM), estrone-3-sulphate (100 µM), and wortmannin (1 µM) were used as influx transport inhibitors. TF3′G (*m/z* 715.1) and ECG (*m/z* 441.1), together with phloretin (*m/z* 273.1) and estrone-3-sulphate (*m/z* 349.1), were visualised via MALDI-MSI in the negative ion-linear mode at a spatial resolution of 50 µm using nifedipine/phytic acid as the matrix reagent. For visualisation of wortmannin (*m/z* 451.1), DHB (acetonitrile/water, 3/1, v/v, 20 mg/mL) was used as the matrix reagent in the positive ion-linear mode. Intensity signals corresponding to each of TF3′G, ECG, estrone-3-sulphate, and wortmannin are shown as fixed pseudocolour scales.

Inhibitor-aided MALDI-MSI was further used to investigate intestinal transport route(s) of TF3′G and ECG in the SD rat jejunum. According to previous report^17^ for investigating transport routes of polyphenols, phloretin (200 µM, an inhibitor of MCT^23^), estrone-3-sulphate (100 µM, an inhibitor of organic anion transporting polypeptides, OATP^24^), and wortmannin (1 µM, an inhibitor of the transcytosis transport pathway^25^) were used in this study for 60-min transport of 50 µM TF3′G and ECG across the SD rat jejunum membrane. MALDI-MSI-guided visualisation of TF3′G (Fig. 2a) showed that both phloretin and estrone-3-sulphate significantly impaired the detection of TF3′G and the local visualisation of each inhibitor at the apical side. MSI results also indicated the first finding that non-absorbable TF3′G (Fig. 1d) was incorporated into the intracellular side of the intestinal membrane. In contrast, we observed no changes in the localisation of TF3′G using wortamannin (Fig. 2a) compared to TF3′G alone, thereby suggesting that incorporation into the membranes did not occur via the transcytosis route. Similar observations on ECG localisation in the membranes were made using each inhibitor (Fig. 2b). The above findings clearly indicated that both TF3′G and ECG can be incorporated into the SD rat jejunum membrane via transporters of the MCT and OATP routes.

**Figure 3:**
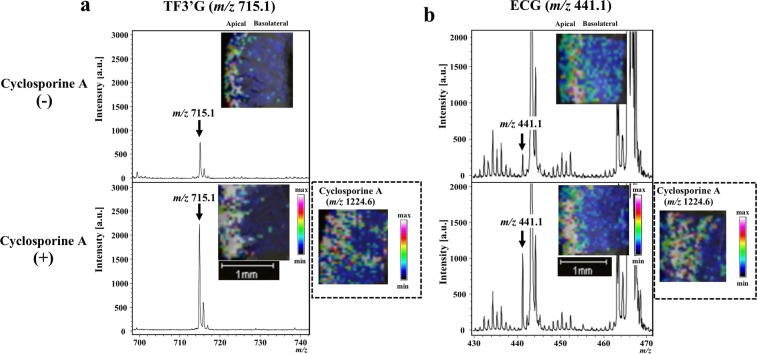
MALDI-MSI visualisation of TF3′G and ECG in jejunum membranes subjected to 60-min transport experiments in the absence or presence of efflux transport inhibitor. Cyclosporine A (20 µM) was used as the efflux transport inhibitor. TF3′G (*m/z* 715.1) and ECG (*m/z* 441.1) were visualised via MALDI-MSI in the negative ion-linear mode at a spatial resolution of 50 µm using nifedipine/phytic acid as the matrix reagent. For visualisation of cyclosporine A (*m/z* 1224.6), DHB (acetonitrile/water, 3/1, v/v, 20 mg/mL) was used as the matrix reagent in the positive ion-linear mode. Intensity signals corresponding to TF3′G, ECG, and cyclosporine A are shown as fixed pseudocolour scales.

Considering that both polyphenols were incorporated into the intestinal membrane, we next performed inhibitor-aided MALDI-MSI to verify whether the incorporated polyphenols were pumped out into the apical compartment. We used cyclosporine A (20 µM), a non-specific inhibitor of efflux transporters, which include ATP-binding cassette (ABC) transporters, such as multidrug resistance protein 2 (MRP2), P-glycoprotein (P-gp), and breast cancer resistance protein (BCRP)^26,27^. As shown in Fig. 3a, cyclosporine A significantly improved the visualisation of TF3′G relative to TF3′G alone. In addition, cyclosporine A increased the intensity of detected ECG (Fig. 3b). The above findings strongly suggested that the non-absorbable TF3′G was effluxed back via ABC transporters into the apical compartment after incorporation into intestinal membrane, whereas absorbable ECG (Fig. 1e) was partially pumped out into the apical compartment.

### MALDI-MSI analysis of TF3′G and ECG metabolites during absorption

**Figure 4:**
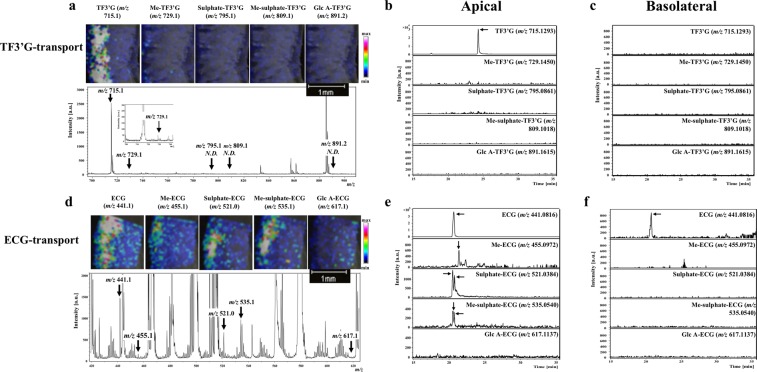
MALDI-MSI analyses of the metabolites TF3′G and ECG in jejunum membranes subjected to 60-min transport experiments in the presence of 20 µM cyclosporine A. TF3′G (*m/z* 715.1) and its metabolites [Me-TF3′G (*m/z* 729.1), sulphate-TF3′G (*m/z* 795.1), Me-sulphate-TF3′G (*m/z* 809.1), Glc A-TF3′G (*m/z* 891.2)] were visualised via MALDI-MSI in the negative ion-linear mode at a spatial resolution of 50 µm using nifedipine/phytic acid as the matrix reagent (**a**). ECG (*m/z* 441.1) and its metabolites [Me-ECG (*m/z* 455.1), sulphate-ECG (*m/z* 521.0), Me-sulphate-ECG (*m/z* 535.1), Glc A-ECG (*m/z* 617.1)] were visualised via MALDI-MSI in the negative ion-linear mode at a spatial resolution of 50 µm using nifedipine/phytic acid as the matrix reagent (**d**). Intensity signals corresponding to TF3′G, ECG, and their metabolites are shown as fixed pseudocolour scales. LC-TOF-MS chromatograms of TF3′G and its metabolites in the apical (**b**) and the basolateral solutions (**c**) were obtained after 60-min transport experiments using the SD rat jejunum membrane. LC-TOF-MS chromatograms of ECG and its metabolites in the apical (**e**) and the basolateral solutions (**f**) were obtained after 60-min transport experiments using the SD rat jejunum membrane. Detailed LC-TOF-MS conditions are described in the Methods section.

**Figure 5:**
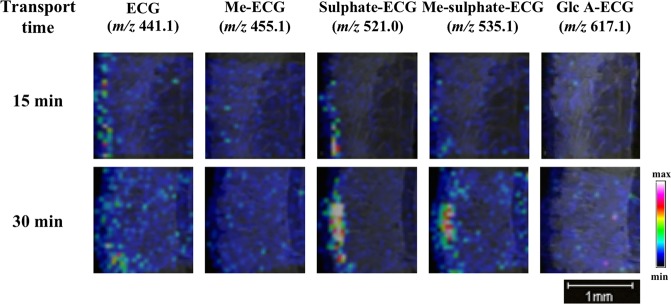
MALDI-MSI visualisation of ECG and its metabolites in the SD rat jejunum membrane at transport periods of 15 and 30 min in the presence of 20 µM cyclosporine A. ECG (*m/z* 441.1) and its metabolites [Me-ECG (*m/z* 455.1), sulphate-ECG (*m/z* 521.0), Me-sulphate-ECG (*m/z* 535.1), Glc A-ECG (*m/z* 617.1)] were visualised via MALDI-MSI in the negative ion-linear mode at the spatial resolution of 50 µm using nifedipine/phytic acid as the matrix reagent. Intensity signals corresponding to ECG and metabolites are shown as fixed pseudocolour scales.

The advantage of MALDI-MSI for non-targeting analysis was further applied to investigate TF3′G and ECG metabolites during the absorption process during 60-min transport experiments using the SD rat jejunum membrane. Cyclosporine A was added to the apical solution to enhance accumulation and MS detection of the target metabolites. Under the present MALDI-MSI conditions and within the MS detection range of *m/z* 100–1000, we detected no TF3′G metabolites that were susceptible to phase II metabolic processes, such as sulphation ([sulphate-TF3′G-H]^−^, *m/z* 795.1), methyl-sulphation ([Me-sulphate-TF3′G-H]^−^, *m/z* 809.1), and glucuronidation ([Glc A-TF3′G-H]^−^, *m/z* 891.2) (Fig. 4a). Although a weak signal corresponding to methylated TF3′G ([Me-TF3′G-H]^−^, *m/z* 729.1) was detected at the apical region, results based on MALDI-MSI (Fig. 4a) and LC-TOF-MS (Fig. 4b,c) strongly suggested that TF3′G (or probably TFs) was stable or less susceptible to phase II metabolism during the influx/efflux absorption process. By contrast, our MALDI-MSI technique clearly demonstrated that absorbable ECG was susceptible to phase II metabolism during intestinal absorption (Fig. 4d). Furthermore, LC-TOF-MS analysis indicated the presence of possible ECG metabolites in the apical solution (Fig. 4e). Methylated ([Me-ECG-H]^−^, *m/z* 455.1), sulphated ([sulphate-ECG-H]^−^, *m/z* 521.0), and methyl-sulphated ECG forms ([Me-sulphate-ECG-H]^−^, *m/z* 535.1) were successfully detected, while glucuronide ECG ([Glc A-ECG-H]^−^, *m/z* 617.1) was not detected (Fig. 4d). Two peaks corresponding to sulphated ECG ([M-H]^−^, *m/z* 521.0384) and methyl-sulphated-ECG ([M-H]^−^, *m/z* 535.0540) in the LC-TOF-MS chromatograms (Fig. 4e) suggested that at least two isomers with different sulphated positions in ECG skeleton were produced during the absorption process. Given that LC-TOF-MS detected these ECG metabolites in the apical solution (Fig. 4e), but not in the basolateral solution (Fig. 4f) after 60-min transport experiments, we speculated that most ECG metabolites were pumped out into the apical compartment after phase II metabolism. Further experiments must be needed to quantify each ECG metabolite by LC-TOF-MS, and are in progress by using rat intestine in terms of transport time. The proposed MALDI-MSI technique indicated rapid metabolism of the incorporated ECG into the sulphated and methyl-sulphated forms occurred in the SD rat jejunum during the transport periods of 15, 30, and 60 min (Figs 4d and 5).

## Discussion

The present study highlighted the potential use of MALDI-MSI as a novel approach for pharmacological studies; in turn, MALDI-MSI has advantage as non-targeting analysis based on *m/z*, providing direct evidence on the location of interesting analytes in tissues without chromatographic separation by LC-MS. The nifedipine/phytic acid-aided MALDI-MSI technique is an *in situ* visualisation technique that successfully and clearly verified the location, transport route(s), and metabolism of the target polyphenols, namely, absorbable ECG and non-absorbable TF3′G, in the SD rat jejunum.

To confirm the validity of the proposed MALDI-MSI technique for absorption studies, we selected ECG, a known absorbable polyphenol^13^, as the target for transport experiments in the SD rat jejunum membrane. Caco-2 cell transport experiment results suggested that ECG was a transportable polyphenol in its intact form^17^. The intact absorption of ECG was also confirmed in SD rats experiments^15^. Results based on our proposed MALDI-MSI visualisation technique were consistent with those of previous reports showing that ECG was absorbed in its intact form, as evidenced by its visualised distribution across the membrane in the SD rat jejunum after 60-min transport experiments (Fig. 1c). Although the current MALDI-MSI visualisation technique can be useful for direct monitoring of targets localised in intestinal tissues (Fig. 1c), the less quantitative characteristics of MALDI-MSI targets will be overcome by the use of LC-MS in future study. The analytical validity of MALDI-MSI was also confirmed by inhibitor-aided technique, which was used to verify ECG transport route(s). Previous studies reported that ECG was transported via the MCT and OATP transporters in Caco-2 cell monolayers^17^ and OATP-transfected cells^28^. In the present study, MALDI-MSI in the presence of an MCT- or OATP- specific inhibitor reduced the visualisation of ECG (Fig. 2b). Our findings showed the reliability of our proposed inhibitor-aided MALDI-MSI technique for direct analysis of absorption behaviour without the requirement of additional analytical steps, such as extraction and separation.

Given the advantages of the MALDI-MSI technique and considering that no studies have reported the distribution of theaflavins (TFs) in their non-absorbable forms in the intestinal membrane, we next attempted to visualise TFs in the SD rat jejunum. MALDI-MSI was successfully used to visualise the region containing TF3′G, which was locally distributed at the apical region (Fig. 1b). Our study is the first to show that TF3′G was not transported across the jejunum membrane, but was incorporated into the apical side of the membrane. Kondo *et al*.^20^ reported that TFs were recognised by renal OATP transporters and that cellular accumulation of TFs was inhibited by treatment of human embryonic kidney cells with OATP inhibitor (estrone-3-sulphate). Thus, in the present study, we utilised estrone-3-sulphate and phloretin (MCT inhibitor) for inhibitor-aided MALDI-MSI analysis to verify the incorporation route (s) of TF3′G into the SD rat jejunum membrane. As shown in Fig. 2a, it was clearly visualised by MALDI-MSI that in the presence of inhibitors, TF3′G was transported by both MCT and OATP transporters, as indicated by the reduced size of the visualisation region, although the involvement of other intestinal route(s) in TF3′G transport was not ruled out. The inhibitor-aided MALDI-MSI technique was used to further investigate the behaviour of TF3′G incorporated in the intracellular jejunum membrane. The results clearly indicated that cyclosporine A, a non-specific inhibitor of ABC transporters, enhanced the visualisation of incorporated TF3′G (Fig. 3a), thereby indicating that TF3′G is exposed to efflux routes, such as MRP2, P-gp, and BCRP, after incorporation into the intracellular apical region. Our further MALDI-MSI experiments using a specific inhibitor targeting each efflux transporter can aid in the determination of the efflux route responsible for pumping out TF3′G.

Polyphenols exhibit poor bioavailability^26^ and are susceptible to phase II metabolic processes, such as methylation, sulphation, and glucuronidation, in the intestines, liver, and kidneys^2,29^. However, the regions responsible for transporting polyphenols into the intestinal membrane have not been identified. For non-absorbable TFs, no phase II-related TF metabolites were detected in human urine after the intake of 1 g of TF extract^30^. Consistent with the abovementioned findings, metabolites were not detected in the membrane (Fig. 4a) and in the apical solution (Fig. 4b) during the 60-min transport experiments of 50 µM TF3′G across the SD rat jejunum membrane. Although the production of TF3′G metabolites cannot be ruled out due to the limitation of MALDI-MSI and LC-MS detection capacities, it is likely that phase II metabolism of TFs is poor in the rat jejunum within the present experimental conditions. In contrast, ECG has been reported to be susceptible to phase II metabolism, thereby, forming methylated, sulphated, and glucuronidated conjugates after a single oral administration in Wistar rats^16^. Other studies also indicated that catechins are preferentially methylated or sulphated during their intestinal absorption in Caco-2 cell monolayers^31^ and small intestine of Wistar rats^29^. Consistent with the findings of previous reports, ECG, a mono-catechin, was metabolised to methylated-, sulphated-, methyl-sulphated-, and to some extent, glucuronide conjugates during the absorption process in the SD rat jejunum membrane (Fig. 4d)^16,29,31^. Another advantage of MALDI-MSI is that it allows non-targeting visualisation, provided novel information that the aforementioned phase II metabolism of ECG occurred rapidly at the same location where the intact ECG is present in the jejunum (Figs 4d and 5).

**Figure 6:**
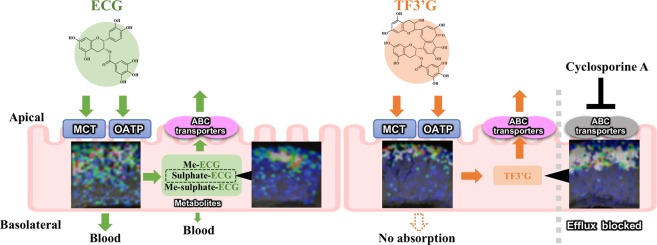
Proposed absorption process of TF3′G and ECG in the SD rat jejunum based on results obtained using the currently proposed inhibitor-aided MALDI-MSI visualisation technique. The inserted ion images were the same as in Figs 1, 3, and 4.

In conclusion, the currently proposed nifedipine/phytic acid-aided MALDI-MSI technique provides the first *in situ* evidence of the location, influx/efflux routes, and metabolism of non-absorbable and absorbable polyphenols (Fig. 6). The visualised distribution of TF3′G in the apical region of SD rat jejunum membrane showed that TF3′G was stable against phase II metabolism during MCT and OATP incorporation/ABC efflux transport processes. In contrast, the non-targeted visualisation by MALDI-MSI indicated the rapid phase II metabolism of ECG to form methylation, sulphation, and their conjugates throughout the jejunum membrane. The visualised localisation of TF3′G and ECG and their metabolites were of huge importance for the elucidation of intestinal absorption mechanisms, since such spatial information would be lost in LC-MS based technique during sample preparation. Therefore, the present *in situ* MALDI-MSI technique can serve as a powerful and novel analytical tool for investigating intestinal absorption and metabolism of polyphenols including flavonoids and phenolic acids without the need for antibodies for immune staining visualisation, and without requiring tedious analytical extraction and separation steps.

## Methods

### Materials

Nifedipine, *trans*-3-indoleacrylic acid (IAA), theaflavin-3′-*O*-gallate (TF3′G), epicatechin gallate (ECG), and phloretin were obtained from Wako Pure Chemical Ind. (Osaka, Japan). 9-aminoacridine (9-AA) was purchased from Merck Millipore (Darmstadt, Germany). 1,5-diaminonaphtalene (1,5-DAN) was obtained from Tokyo Chemical Ind. (Tokyo, Japan). 2,5-dihydroxybenzoic acid (DHB) and estrone-3-sulphate were purchased from Sigma-Aldrich (St. Louis, MO, USA). Cyclosporine A and phytic acid were obtained from Nacalai Tesque Co. (Kyoto, Japan). Wortmannin was purchased from Enzo Life Science (Lausen, Switzerland). All other chemicals were of analytical reagent grade and were used without further purification.

### Transport experiments using the rat intestinal membrane in the Ussing Chamber system

Intestinal transport experiments using SD rat jejunum membranes was performed according to our previous report^4^. After 16 h of fasting, a segment of the jejunum (15–20 cm below the stomach) was removed from the small intestine of nine-week-old male SD rats (SPF/VAF Crj:SD; Charles River Japan, Kanagawa, Japan) and subsequently washed with Krebs-Bicarbonate Ringer’s solution (KBR, pH 7.4, 2.5 mM CaCl_2_, 4.8 mM KCl, 1.3 mM KH_2_PO_4_, 1.2 mM MgSO_4_, 118.1 mM NaCl, 10 mM d-glucose, and 25 mM NaHCO_3_). The jejunum was cut along the mesenteric border to expose the mucosal side and subsequently mounted onto the Ussing Chamber system (Dual Channel Ussing Chamber, model U-2500, Warner Instrument, Hamden, CT, USA). KBR solution (5 mL) was added to the apical (pH 6.0) and basolateral (pH 7.4) sides. After 15 min of incubation, the apical solution was replaced with fresh KBR buffer containing 50 µM TF3′G or ECG. For the inhibitor experiments, fresh KBR solution containing the inhibitor dissolved in 0.05% DMSO (20 µM cyclosporine A, 200 µM phloretin, 100 µM estrone-3-sulphate, or 1 µM wortmannin) was added to the apical side. After 15 min of incubation, KBR buffer containing the inhibitor and 50 µM TF3′G or ECG were added to apical side, after which transport experiments were conducted for 60 min. During the transport period, a 95:5 O_2_/CO_2_ mixture was continuously bubbled through the apical and basolateral solutions through air vents in the chamber to improve tissue viability and provide stirring during transport. After 60 min of transport, the apical and basolateral solutions were collected for subsequent LC-TOF-MS. The intestinal membranes were washed thrice with KBR buffer and immediately frozen in powdered dry ice. The quickly frozen membranes were stored at −30 °C until MALDI-MSI. All animal experiments were carried out in accordance with the guidelines set by the Guidance for Animal Experiments in the Faculty of Agriculture and in the Graduate Course of Kyushu University in accordance with the Law (No. 105, 1973) and Notification (No. 6, 1980, of the Prime Minister’s Office) of the Japanese Government. All experimental protocols were reviewed and approved by the Animal Care and Use Committee of Kyushu University (permit number: A28-040).

### LC-TOF-MS analysis

After the 60-min transport experiments, an aliquot (4 mL) of the apical or basolateral side was added to a Waters Sep-Pak Plus C_18_ cartridge (Waters, Milford, MA, USA) and eluted with methanol (MeOH)/formic acid (FA) (100/0.1, v/v). The eluate was evaporated to dryness and dissolved in 100 µL of MeOH/water/FA (50/50/0.1, v/v/v). Next, 20 µL of the solution was injected into the LC-TOF-MS system. Separation of TF3′G, ECG, and metabolites was performed using an Agilent 1200 series HPLC (Agilent Technologies, Waldbronn, Germany) equipped with a micro degasser (G1379B), binary pump (G1312A), a thermostatically controlled oven compartment (G1316A), and a Cosmosil 5C_18_-MS-II column (2.0 mm × 150 mm, Nacalai Tesque Co.). The mobile phase containing solvent A (water/FA, 100/0.1, v/v) and solvent B (MeOH/FA, 100/0.1, v/v) was used to establish a 30-min linear gradient from 0% to 100% of solvent B, at a flow rate of 0.2 mL/min at 40 °C. MS analysis was performed using a micrOTOF-II mass spectrometer (Bruker Daltonics, Bremen, Germany). Ionisation was performed via electrospray ionisation (ESI) in the negative mode, and the mass range was set to *m/z* 100–1000. The ESI-MS conditions were set as follows: drying gas (nitrogen) at flow rate of 8.0 L/min; drying temperature, 200 °C; nebulising gas pressure, 1.6 bar; and capillary voltage, 3800 V. Calibration was performed at the beginning of each run using a sodium formate solution containing 10 mM sodium hydroxide in water/acetonitrile (1/1, v/v). Data acquisition and analysis were carried out using the Bruker Data Analysis 3.2 software.

### Preparation of rat intestinal membrane sections for MALDI-MSI

The frozen intestinal segment was sliced into 12-µm-thick sections at the cross-sectional face using a CM1100 Leica Cryomicrotome (Leica, Wetzler, Germany) at −20 °C. Intestinal sections were thaw-mounted on an indium-tin oxide (ITO)-coated conductive glass slide (Bruker Daltonics) and subsequently dried under nitrogen gas flow.

An ImagePrep automatic matrix sprayer (Bruker Daltonics) was used to spray the matrix uniformly over the ITO glass slide. The following matrix reagents were used: IAA, 1,5-DAN, DHB, and nifedipine at 20 mg/mL in acetonitrile/water (3:1, v/v); 9-AA at 10 mg/mL in acetonitrile/water (3:1, v/v); and nifedipine at 20 mg/mL in acetonitrile/water (3:1, v/v) containing 5 mM phytic acid. The following spraying conditions were used: spray power, 20%; modulation, 20%; spraying time, 1.5 sec; incubation time, 10 sec; drying time, 60 sec; and spraying, 60–70 cycles.

### MALDI-MSI analysis

MALDI-MSI was performed using an Autoflex III mass spectrometer equipped with a SmartBeam III (Bruker Daltonics). MALDI-MSI in the negative ion-linear mode was performed to analyse TF3′G, ECG, and their metabolites, as well as phloretin and estrone-3-sulphate. For cyclosporine A and wortmannin detection, MS data were acquired within the range of *m/z* 100–1300 in the positive ion-linear mode. The MS parameters were as follows: ion source 1, 20.00 kV; ion source 2, 18.80 kV; lens voltage, 7.50 kV; gain, 12.00; laser frequency, 200 Hz; laser power 80%; off set, 60%; range, 20%; laser focus range, 100%; and value, 6%. MSI analysis was performed with a spatial resolution of 50 µm. The acquired MS spectra were analysed using Bruker FlexAnalysis software (version 3.3) as the sum of randomly selected 100 spectra in the region of MS imaging shown in Fig. 1a. Ion image data were reconstructed for visualisation with a mass filter of ± 0.2 *m/z* using the Bruker FlexImaging software (version 2.1). In this study, we used observed MS intensity of targets to evaluate the difference in MSI visualisation between tissue sections without normalisation. The current MALDI-MSI assay had good reproducibility and reliability owing to the formation of homogenous matrix crystals by phytic acid^4^. Indeed, the MS signal of nifedipine sprayed on different sections was constant ([M-H]^−^ *m/z* 345.1, intensity: 4.15 ± 0.51 × 10^5^, CV: 13%, Fig. S1).

## Supplementary information

Supplemental Figure S1

## Data Availability

All data generated or analysed during this study are included in this published article.
