# Supplementary material for: Novel in situ visualisation of rat intestinal absorption of polyphenols via matrix-assisted laser desorption/ionisation mass spectrometry imaging
Source: Sci Rep. 2019 Feb 28;9:3166. doi: 10.1038/s41598-019-39405-w (PMC6395804; doi:10.1038/s41598-019-39405-w)
Supplement: Supplementary file 1 — Supplemental Figure S1 [file 41598_2019_39405_MOESM1_ESM.pdf]

Supplementary Information

Novel *in situ* visualisation of rat intestinal absorption of polyphenols via matrix-assisted laser desorption/ionisation mass spectrometry imaging

Huu-Nghi Nguyen[1#], Mitsuru Tanaka[1#], Baorui Li[1], Tomoya Ueno[2], Hideki Matsuda[2] and Toshiro Matsui[1*]

[1] Division of Bioscience and Bioenvironmental Sciences, Faculty of Agriculture, Graduate School of Kyushu University, 744 Motooka, Nishi-ku, Fukuoka, 819-0395, Japan

[2]Division of Research and Development, Yaizu Suisankagaku Ind. Co. Ltd., 5-8-13 Kogawashinmachi, Yaizu, Shizuoka, 425-8570, Japan

[#] These authors contributed equally.

*Correspondence to: Toshiro Matsui, Division of Bioscience and Bioenvironmental Sciences, Faculty of Agriculture, Graduate School of Kyushu University, 744 Motooka, Nishi-ku, Fukuoka, 819-0395, Japan

E-mail: tmatsui@agr.kyushu-u.ac.jp

Fax: +81-92-802-4752

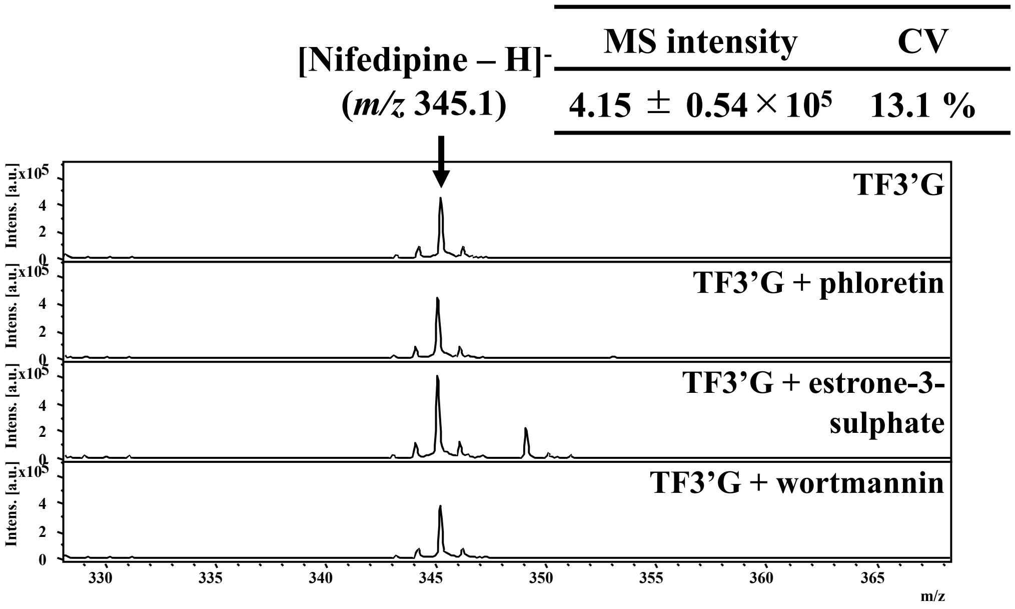

Figure S1. MALDI-MS spectra of nifedipine ($[M\text{-}H]^-$ *m/z* 345.1) on jejunum membrane sections from 60-min transport experiments of TF3'G. The detail experimental conditions were described in Method section. The phytic acid-aided homogenous matrix crystal sprayed by an ImagePrep showed constant MS intensity of nifedipine (intensity: $4.15 \pm 0.51 \times 10^5$, CV: 13%) between different tissue sections.
